# Menopausal symptoms, attitudes toward menopause, and social media/internet use among women in the climacteric period: a cross-sectional study

**DOI:** 10.1590/1980-220X-REEUSP-2026-0107en

**Published:** 2026-08-14

**Authors:** Nazlı Emel Özer Yurdal, Emine Gerçek Öter, İbrahim Eren Pek, Mehmet Nuri Duran

**Affiliations:** 1 Çanakkale Onsekiz Mart University, Research Hospital, Obstetrics and Gynaecology Nursing, Çanakkale, Turkey.; 2 Aydın Adnan Menderes University, Faculty of Nursing, Department of Obstetrics and Gynecology Nursing, Aydın, Turkey.; 3 Çanakkale Onsekiz Mart University, Faculty of Medicine, Obstetrics and Gynaecology, Çanakkale, Turkey.; 4 Ministry of Health Ezine State Hospital, Department of Obstetrics and Gynaecology, Çanakkale, Turkey.

**Keywords:** Climacteric, Health Literacy, Internet, Menopause, Nursing, Social Media, Climaterio, Alfabetización en Salud, Internet, Menopausia, Enfermería, Medios de Comunicación Sociales

## Abstract

**Objective:**

To examine associations between menopausal symptom severity, attitudes toward menopause, and internet/social media use among climacteric women.

**Method:**

This analytical cross-sectional study included 250 women aged 40–64 years attending gynecology inpatient units and family medicine outpatient clinics at a university hospital. Data were collected between July and September 2025, after ethics committee approval. Participants completed questionnaires including validated scales for menopausal attitudes and symptom severity.

**Results:**

Most participants were postmenopausal (61.2%); mean age was 49.2 years. Internet/social media use indicators were not associated with attitudes toward menopause. Higher severity in symptoms was associated with longer daily internet/social media use, obtaining menopause-related information online, and searching online before medical consultation. A moderate general internet/social media use pattern (2–3 times/week and 1–3 hours/day) was associated with lower symptom severity.

**Conclusion:**

Online information-seeking was associated with menopausal symptom severity but not attitudes. Nurses and other health professionals should assess online information-seeking and support digital health literacy by guiding women to reliable resources.

## RESUMO

**Objetivo:**

Examinar as associações entre a gravidade dos sintomas da menopausa, as atitudes em relação à menopausa e o uso de internet/mídias sociais em mulheres no climatério.

**Método:**

Estudo transversal analítico que incluiu 250 mulheres de 40 a 64 anos atendidas em unidades de internação de ginecologia e em ambulatórios de medicina de família de um hospital universitário. Os dados foram coletados entre julho e setembro de 2025, após aprovação do comitê de ética. As participantes responderam a questionários que incluíam escalas validadas para atitudes em relação à menopausa e gravidade dos sintomas.

**Resultados:**

A maioria das participantes era pós-menopáusica (61,2%); a média de idade foi de 49,2 anos. Os indicadores de uso de internet/mídias sociais não se associaram às atitudes em relação à menopausa. A maior gravidade dos sintomas associou-se a maior tempo diário de uso de internet/mídias sociais, à obtenção de informações sobre a menopausa on-line e à busca on-line antes da consulta médica. Um padrão moderado de uso geral de internet/mídias sociais (2 a 3 vezes por semana e 1 a 3 horas por dia) associou-se a menor gravidade dos sintomas.

**Conclusão:**

A busca de informações on-line associou-se à gravidade dos sintomas da menopausa, mas não às atitudes. Enfermeiros e outros profissionais de saúde devem avaliar a busca de informações on-line e apoiar o letramento digital em saúde, orientando as mulheres para fontes confiáveis.

## INTRODUCTION

Menopause is a natural life stage and it is clinically defined as the permanent cessation of menstruation, diagnosed retrospectively after 12 consecutive months of amenorrhoea in the absence of other pathological or physiological causes^(1)^. The climacteric period is broader than menopause and includes the late reproductive years, the menopausal transition, and early post-menopause. Although the STRAW+10 staging system provides a standardized framework for reproductive aging, the present study has considers it solely as a conceptual framework for defining the climacteric period and does not use it as an analytical staging criterion^(2)^.

The menopausal transition is frequently accompanied by vasomotor symptoms, sleep disturbances, mood and cognitive complaints, and genitourinary symptoms, which may substantially impair quality of life and daily functioning^(1,3)^. Hormone therapy remains the most effective option for managing vasomotor symptoms and the genitourinary syndrome of menopause when not contraindicated, and treatment decisions should be individualized^(3)^. Beyond biological changes, women’s attitudes and expectations toward menopause play an important role in how symptoms are perceived and reported; evidence suggests that more negative attitudes are often associated with higher symptom reporting, whereas more positive attitudes may buffer the symptom experience^(4)^.

In parallel, the internet and social media have become major channels through which women seek health information. Social media is commonly defined as a group of internet-based applications that build on the ideological and technological foundations of Web 2.0 and allow the creation and exchange of user-generated content^(5)^. Studies show that many menopausal women actively search online for information about symptoms, management options and peer experiences^(6)^. However, the online environment also facilitates the rapid spread of incomplete, misleading or commercially biased content—an “infodemic”—which can make it difficult for individuals to identify trustworthy guidance and may influence health beliefs and care-seeking behaviours^(7)^.

Türkiye is undergoing demographic ageing; according to the 2024 Address Based Population Registration System results, the median age in Türkiye increased to 34.4 years^(8)^. Çanakkale province is among the regions with an older age structure, with a median age of 41.7 years in 2024 (40.8 for men and 42.6 for women)^(9)^. This profile approaches the median age reported for the European Union (44.7 years on 1 January 2024)^(10)^. Therefore, studying menopause-related experiences in Çanakkale province may provide insights relevant to settings with comparatively older population structures.

Given the growing role of digital information sources, menopause counselling should also incorporate support for digital health literacy (eHealth literacy), which refers to individuals’ perceived skills in finding, evaluating and applying electronic health information to health problems^(11)^. Within this context, this cross-sectional study aims to examine whether patterns of internet/social media use—particularly menopause-related online information seeking—were associated with menopausal symptom severity and attitudes toward menopause among women in the climacteric period. Specifically, the study aims to (1) describe the level of menopausal symptom severity; (2) describe women’s attitudes toward menopause; (3) assess associations between internet/social media use and both symptom severity and attitudes; and (4) characterize women’s general internet/social media use patterns and menopause-related online information-seeking behaviors.

## METHODS

### Study Design

The design used is an analytical cross-sectional methodology.

### Ethical Considerations

Written informed consent to participate in the study was obtained from all participants in accordance with the Declaration of Helsinki. Ethics committee approval was obtained from Çanakkale Onsekiz Mart University Clinical Research Ethics Committee with the 2025-07/07-02 decision and protocol number 2025/97 dated 17.04.2025. Institutional permission was obtained from the relevant hospital units before the data collection process. Subsequently, written permission to collect data was obtained from the Chief Physician’s Office of Çanakkale Onsekiz Mart University Hospital.

### Setting and Participants

The study was conducted at the gynecology inpatient unit and family medicine outpatient clinics of Çanakkale Onsekiz Mart University Hospital. Data were collected between July 2025 and September 2025, after ethics committee approval had been obtained.

### Sample Size

According to clinic records, approximately 1,500 women aged ≥40 years attend these clinics annually. An a priori sample size calculation was performed using G*Power 3.1. Assuming a medium effect size (Cohen’s d = 0.50), an alpha level of 0.05, and 95% power (1–β = 0.95), the minimum required sample size was 202 participants. To compensate for potential nonresponse and incomplete data, the target sample size was set at 250 women.

### Eligibility Criteria

#### Inclusion Criteria

- aged 40–64 years;
- willing to participate and provided informed consent signature;
- no diagnosed psychiatric disorder;
- not using psychiatric medication; and
- not using any medication for menopausal symptoms.

#### Exclusion Criteria

- withdrawal of consent or unwillingness to continue participation; and
- incomplete questionnaires or technical problems during data collection.

### Data Collection Tools

The study data were collected using a three-part questionnaire: (1) a Personal Information Form querying women’s sociodemographic/biographical characteristics and menopause-related history, (2) the Menopause Attitude Assessment Scale (MAAS—Turkish form), and (3) the Menopause Rating Scale (MRS—Turkish form)^(12,13,14,15,16,17,18)^.

### Questionnaire Form

The questionnaire was prepared by the researchers, following the literature^(13,14,15,16,17,18,19,20,21,22)^. It comprised 26 items designed to obtain women’s sociocultural and demographic characteristics (e.g., age, education, economic status), menopause-related characteristics, and digital/social media use characteristics (e.g., daily social media and internet use, use of the internet or social media to obtain menopause-related information, and online searches before consulting a physician).

The item on weekly internet/social media use assessed participants’ general use of social media and the internet, not menopause-specific content use. This item included three frequency categories (once per week, 2–3 times per week, and 6–7 days per week) and asked participants to report their approximate daily duration of use. For analysis, the reported daily duration was categorized as <1 hour, 1–3 hours, and ≥4 hours. The category “2–3 times per week and 1–3 hours per day” was therefore constructed by combining the weekly frequency category “2–3 times per week” with the daily duration category “1–3 hours per day.” This variable was interpreted as a moderate general internet/social media use pattern and not as menopause-specific engagement or a protective exposure.

The draft questionnaire (including the Personal Information Form and items on social media/internet use) together with the scale administration instructions was sent to 20 experts to assess content validity and clarity. The expert panel was planned to include faculty and clinical nurses in Women’s Health and Obstetrics Nursing, specialists in Obstetrics and Gynecology, Family Medicine and Public Health, and one expert in measurement–evaluation/psychometrics. Each item was rated on a three-point scale: “Not appropriate (0)” / “Appropriate but needs revision (1)” / “Appropriate (2)”. Of the 20 invited experts, 12 responded; Krippendorff’s alpha = 0.833 indicated a high level of inter-expert agreement. Language and content revisions were made based on expert feedback, and the final version of the form was produced. This form provided as Supplementary file 1.

*Pilot testing of the developed questionnaire form:* A near-final version was piloted with 8 menopausal women selected from the target population. The pilot showed that items were understandable, instructions adequate, and the completion time feasible; therefore, full data collection proceeded. Data from the pilot were not included in the final analysis.

### Menopause Attitude Assessment Scale (MAAS)

Originally developed in 1994 to measure attitudes toward menopause and the postmenopausal period across different age groups in Türkiye, the scale contains 2 positive and 18 negative statements. Positive items are scored 0 (“strongly disagree”) to 4 (“strongly agree”); negative items are reverse scored. Possible total scores range from 0 to 80; higher scores indicate a more positive attitude toward menopause, whereas lower scores indicate a negative attitude. Scores above the mean reflect increasingly positive attitudes^(14)^. The reliability of the scale was reported as 0.86^(15)^. This form provided as Supplementary file 2.

### Menopause Rating Scale (MRS—Turkish Form)

The MRS, originally developed in 1992 and adapted into Turkish in 2005, consists of 11 items, three subscales, and a 5-point Likert format (none, mild, moderate, severe, very severe). Total scores range from 0 to 44. The three subscales are: Somatic (items 1, 2, 3, 11), Psychological (items 4, 5, 6, 7), and Urogenital (items 8, 9, 10). An increase in the total score reflects an increase in the severity of symptoms and a deterioration in quality of life (16, 19). The Cronbach’s alpha reliability coefficient of the Turkish form is 0.84^(16,19)^. This form provided as Supplementary file 3.

### Data Collection

Data were collected between July 2025 and September 2025 at the gynecology inpatient unit and family medicine outpatient clinics of Çanakkale Onsekiz Mart University Hospital. On days when the researchers were available (including weekdays, outside working hours, and weekends), eligible women were approached consecutively and informed about the study purpose, procedures, confidentiality, and their right to refuse or withdraw at any time without consequences. Written informed consent was obtained prior to participation.

Participants completed the questionnaires using a self-administered (self-report) format in a suitable area that allowed privacy. A trained researcher remained available during completion to clarify procedural questions (e.g., how to mark responses) and to ensure that forms were completed individually, but did not guide or influence participants’ answers. Completion took approximately 15 to 20 minutes. After submission, questionnaires were checked on-site for missing responses; if any items were unintentionally left blank, participants were asked whether they wished to complete them. Forms with substantial missing data or affected by technical/administrative problems were excluded in line with the exclusion criteria. Recruitment continued until the target sample size was reached.

### Data Analysis

Data were analysed using the JAMOVI statistical package (version 2.6.44-solid). Normality was assessed with the Kolmogorov–Smirnov and Shapiro–Wilk tests. Descriptive statistics were reported as count, percentage, mean, standard deviation, and minimum–maximum. Relationships between total scale scores were examined using Spearman’s rank correlation (ρ).

### Reporting Guideline

This cross-sectional study was reported in accordance with the STROBE statement; the completed checklist is provided as Supplementary file 4.

## RESULTS

A total of 250 women were analysed. Table 1 presents the demographic characteristics captured in the researcher-developed questionnaire (demographic section), including age (categorised; overall mean ± SD 49.2 ± 6.50), education, employment, marital and economic status, parity, chronic disease, and smoking/alcohol use.

**Table 1:** Distribution of women’s descriptive characteristics (N = 250) – Çanakkale province, Türkiye, 2025.

Variable	n	%
**Age (years)** ^*^		
40–43	59	23.6
44–48	74	29.6
49–53	47	18.8
54–64	70	28.0
**Mean ± SD = 49.2 ± 6.50**		
Education		
High school	69	27.6
Vocational college (two-year associate degree program)	25	10.0
Bachelor’s degree	128	51.2
Postgraduation	28	11.2
**Employment in an income-generating job**		
Yes	192	76.8
No	58	23.2
**Marital status**		
Married	194	77.6
Single	45	18.0
Other	11	4.4
**Economic status**		
Income equals expenses	149	59.6
Income less than expenses	77	30.8
Income greater than expenses	24	9.6
**Children**		
Yes	183	73.2
No	67	26.8
**Chronic disease**		
Present	79	31.6
Absent	171	68.4
**Smoking**		
Yes	92	36.8
No	158	63.2
**Alcohol use**		
Yes	42	16.8
No	208	83.2

*Data were obtained from the researcher-developed questionnaire (26-item instrument).

Values are shown as n (%) unless otherwise indicated; age is presented in categories, and the overall mean ± SD is also provided.

29.6% of the women were in the 44–48 age group; the mean age was 49.2 ± 6.50 years. Overall, 51.2% held a bachelor’s degree, 76.8% were employed in income-generating work, 77.6% were married, and 59.6% reported income equal to expenses. In addition, 73.2% had children, 68.4% reported no chronic disease, 63.2% were non-smokers, and 83.2% reported no alcohol use (Table 1).

Among the participants, 61.2% had entered menopause. Among these women, the mean time since menopause was 3.61 ± 3.98 years. When asked about their feelings at the time of entering menopause, 26.4% reported negative emotions (e.g., sadness, sense of loss) (Table 2).

**Table 2:** Menopause-related characteristics of participants (N = 250) – Çanakkale province, Türkiye, 2025.

Characteristic	n	%
**In the menopause?**		
Yes	153	61.2
No	97	38.8
Time since menopause had begun (years)^*^	**Mean**	**SD**
	3.61	3.98
Feelings at the time they entered menopause^*^		
Negative feelings (sadness, sense of loss, etc.)	66	26.4
No particular feelings	47	18.8
Positive feelings (relief, joy, etc.)	40	16.0

*Answered only by women who had already entered menopause..

Values are n (%) unless otherwise indicated; continuous variable is presented as mean lues are and SD.

Among the women in the study, 88.4% reported using social media and the internet in daily life. The proportion who used these channels to search for information about menopause was 55.6%; among them, 12.4% considered the information reliable, and 15.2% found it useful. In total, 88.0% stated that the online information they obtained did not affect their attitude toward menopause. While 51.6% searched the internet/social media for ways to cope with menopausal symptoms, 84.8% did not find this coping information useful; additionally, 51.6% reported searching online before consulting a physician. Regarding usage patterns, 79.2% used online platforms at least once per week, and 83.2% spent 1–3 hours per day online. The most frequently used platform was the Google search engine (32.4%), and the most common access device was the mobile phone (69.6%). Finally, 33.2% of the women stated that they would like to receive information about menopausal symptoms and coping from nurses/midwives/health professionals, and 28.8% from family physicians (Table 3).

**Table 3:** Social media and internet use among participants (N = 250) – Çanakkale province, Türkiye, 2025.

Item	n	%
**Do you use social media and the internet in daily life?**		
Yes	221	88.4
No	29	11.6
**Do you use social media/the internet for information about menopause?**		
Yes	139	55.6
No	111	44.4
**Do you trust the information obtained online about menopause?**		
Yes	31	12.4
No	219	87.6
**Do you find online information about menopausal symptoms useful?**		
Yes	38	15.2
No	212	84.8
**Do you think what you have learned online affect your attitude toward menopause?**		
Yes	30	12.0
No	220	88.0
Direction of the effect on attitude (n = 30)^*^		
Positive	5	16,7
Negative	25	83,3
**Have you sought information online about coping with menopausal symptoms?**		
Yes	129	51.6
No	121	48.4
**Do you find the information obtained online about coping with symptoms useful?**		
Useful	38	15.2
Not useful	212	84.8
**Do you search information online before visiting a doctor for menopausal symptoms?**		
Yes	129	51.6
No	121	48.4
**Frequency of social media & internet use**		
Once per week	198	79.2
2–3 times per week	48	19.2
6–7 days per week	4	1.6
**Daily time spent on the internet**		
< 1 hour	22	8.8
1-3 hours	208	83.2
4-7 hours and more	20	8.0
Most-used platforms^*^		
Facebook	31	12.4
X (old Twitter)	9	3.6
Instagram	77	30.8
Youtube	48	19.2
Google (Search Engine)	81	32.4
TikTok	4	1.6
Used device(s) for social media^*^		
Mobile phone	174	69.6
Computer	56	22.4
Tablet	20	8.0
**Sources of information on menopausal symptoms and coping**		
School/education	40	16.0
Family physician	72	28.8
Nurse/midwife/health professional	83	33.2
Hospital	30	12.0
Friends	8	3.2
Books/journals/theses/e-journals	1	0.4
Internet sources	6	2.4
Menopause mobile applications	4	1.6
Television	6	2.4

*Multiple responses allowed.

The women’s mean MRS (Turkish form) total score was 16.95 ± 6.67. By subscale, the mean scores were 5.98 ± 2.82 for psychological, 6.76 ± 2.61 for somatic, and 4.20 ± 2.05 for urogenital symptoms (Table 4). The minimum and maximum MAAS (Turkish form) scores were 26 and 80, respectively; the mean total MAAS score was 53.04 ± 10.36 (Table 4).

**Table 4:** Scores on the Menopause Rating Scale (MRS—Turkish form) and Menopause Attitude Assessment Scale (MAAS—Turkish form) (N = 250) – Çanakkale province, Türkiye, 2025.

Scale / subscale	Possible range	Observed Min–Max	Mean ± SD
MAAS total	0–80	26–80	**53.04 ± 10.36**
MRS total	0–44	3–43	**16.95 ± 6.67**
MRS subscales
Psychological symptoms	0–15	0–15	**5.98 ± 2.82**
Somatic symptoms	0–16	0–16	**6.76 ± 2.61**
Urogenital symptoms	0–13	0–13	**4.20 ± 2.05**

* Note. Higher MRS scores indicate greater symptom severity; higher MAAS scores indicate a more positive attitude toward menopause. Values are presented as observed Min–Max and Mean ± SD.

When the relationship between women’s social media/internet use and the MAAS total score was examined, no significant associations were found for daily use of social media/internet, using these channels to search for menopause-related information, trusting the information obtained online, or finding such information useful. By contrast, obtaining information from the internet/social media to cope with menopausal symptoms showed a negative and non-significant relationship with the total MAAS score; searching for menopause-related information and trusting the online information obtained also showed non-significant relationships with the total MAAS score. Additionally, searching the internet or social media before seeing a physician for menopausal symptoms and having a moderate general internet/social media use pattern, defined as using social media/the internet 2–3 times per week and 1–3 hours per day, showed positive but non-significant associations with the total MAAS score (Table 5).

**Table 5:** Spearman correlations between social media/internet use and MAAS/MRS total scores (N = 250) – Çanakkale province, Türkiye, 2025.

Variables	MAAS Total Mean		MRS Total Mean	
	r	p	r	p
Using social media and the internet in daily life	0.058	0.363	0.523	<0.001
Using social media/the internet for information about menopause	0.002	0.975	0.691	<0.001
Trusting the information obtained online about menopause	–0.024	0.711	0.009	0.893
Finding online information on menopausal symptoms useful	0.031	0.628	–0.110	0.083
Seeking information online about coping with menopausal symptoms	–0.044	0.488	–0.117	0.065
Searching online before visiting a doctor for menopausal symptoms	0.092	0.147	0.644	<0.001
Moderate general internet/social media use pattern: 2–3 times per week and 1–3 hours per day	0.059	0.354	–0.237	<0.001

*Note. Correlations are Spearman’s ρ. MAAS = Menopause Attitude Assessment Scale (Turkish form); MRS = Menopause Rating Scale (Turkish form). p < 0.05 considered statistically significant. Correlations are Spearman’s ρ with corresponding p-values.

According to Spearman’s correlation analysis, women’s daily use of social media/internet, using social media/internet for menopause-related information, and searching online before consulting a physician for menopausal symptoms were positively and significantly associated with the MRS total score. In contrast, the moderate general internet/social media use pattern, defined as using social media/the internet 2–3 times per week and 1–3 hours per day, was negatively and significantly associated with the MRS total score. This variable reflects general internet/social media use frequency and duration rather than menopause-specific content use. Finding online information on menopausal symptoms useful and seeking online information about coping with menopausal symptoms showed negative but non-significant correlations with the MRS total score. Trusting online menopause-related information was not significantly associated with the MRS total score. Because of the cross-sectional design, these findings should be interpreted as associations and do not indicate the direction of the relationship.

## DISCUSSION

In this study, we examined whether women’s internet and social media use was associated with attitudes toward menopause and menopausal symptoms. Overall, attitudes toward menopause were not significantly associated with daily internet/social media use, using these channels for menopause-related purposes, searching online before consulting a physician, obtaining online information to cope with menopausal symptoms, trusting the information obtained online, or perceiving it as useful. In contrast, menopausal symptom severity varied across specific online behaviors: daily internet/social media use, using these channels to obtain menopause-related information, and searching online before consulting a physician for menopausal symptoms were positively and significantly associated with the MRS total score, whereas a moderate general internet/social media use pattern (2–3 times per week and 1–3 hours per day) was negatively and significantly associated with the MRS total score. Because this variable was derived from the general internet/social media use item, it should not be interpreted as a measure of menopause-specific engagement. However, the cross-sectional design does not allow determination of whether more severe symptoms led women to seek online information more frequently, or whether exposure to menopause-related online content influenced symptom perception. Therefore, all findings should be interpreted as associations rather than causal effects.

Trust in online menopause information and perceiving online information as useful were not associated with symptom severity. Taken together, these findings suggest that the purpose and pattern of online engagement may matter more than online use alone and highlight the clinical importance of routinely assessing online information-seeking and supporting digital health literacy by guiding women toward reliable resources.

Providing women with planned education about menopause contributes to a positive development in attitudes toward menopause^(23)^. It has also been reported that most women had previously received information about menopause, mainly through television, and that their quality of life was negatively affected^(24)^. Women’s attitudes toward menopause may vary, with some women expressing positive attitudes and others negative attitudes. In addition, a considerable proportion of women may not have sufficient knowledge about menopause and may primarily use social media and the internet to obtain menopause-related information. Media content may sometimes emphasize the negative aspects of menopause while highlighting the positive aspects less frequently, which may increase women’s anxiety and concerns about menopause. Therefore, menopause should not be framed as an excessively medical problem^(25)^. One of the notable findings of this study was the apparent discrepancy between high levels of digital information-seeking and low levels of trust in, and perceived usefulness of, online menopause-related information. Although more than half of the participants reported using the internet or social media to search for information about menopause and searched online before consulting a physician, only a small proportion reported trusting this information or finding it useful. This pattern may indicate that women have unmet information needs during the climacteric period, but may also experience difficulty in evaluating the credibility, accuracy, and applicability of menopause-related online content. For nursing practice, this finding suggests that asking women what they have searched for online, what sources they have used, and whether they trust these sources may be an important component of menopause counselling. Such an approach may help nurses identify misinformation, correct misunderstandings, and guide women toward evidence-based resources.

Women’s daily use of the internet and social media, their use of these channels for menopause-related information and searching online before consulting a physician were positively and significantly associated with menopausal symptom severity. However, the direction of these associations cannot be determined. Women experiencing more severe symptoms may be more likely to search for information online before seeking professional care. Conversely, repeated exposure to incomplete, inconsistent, or anxiety-provoking information may influence how symptoms are perceived. The finding that a moderate general internet/social media use pattern was associated with lower symptom severity should also be interpreted cautiously. This variable reflects general internet/social media use frequency and duration rather than menopause-specific content use. Therefore, rather than indicating a protective effect, this association may reflect differences in general digital behavior, selective information-seeking, or other unmeasured factors such as education, health literacy, or access to reliable information.

Overall, internet/social media use showed no significant association with attitudes toward menopause in our sample, whereas symptom severity varied across online information-seeking behaviours. Recent mobile- and internet-based menopause interventions likewise report reductions in vasomotor and behavioural symptoms among women who engage with digital platforms^(19,20,21)^. In this respect, our findings are in line with the literature. Among women aged ≥40 years, no relationship was found between trusting or finding useful the menopause-related information obtained from the internet/social media and menopausal symptoms.

Some women in the climacteric period may not trust or find useful the information obtained online; accordingly, only a limited proportion have been reported to use the internet to cope with menopausal symptoms^(13)^. Internet-based cognitive behavioural therapy has been shown to reduce hot flushes and improve sleep quality^(26)^. Among postmenopausal and older women, the use of information and communication technologies has also been associated with lower levels of frailty^(22)^. In addition, a digital care/improvement program has been reported to be effective in helping women cope with menopausal symptoms^(19)^. A significant association has been found between health literacy and menopausal symptoms, and improving health literacy in menopausal women has been associated with better quality of life^(27)^. It has also been reported that many women do not recognise menopausal symptoms, need support or professional help, and may benefit from platforms developed for these needs. Gaps in menopause knowledge and literacy have also been identified; furthermore, about half of women preferred a web-based intervention, and most women found an e-health tool based on cognitive behavioural therapy useful and were willing to use it^(28)^. These findings have practical implications for nursing care. Women’s health nurses can incorporate brief questions about online information-seeking into routine menopause consultations, such as whether women have searched for menopause-related information online, which websites or social media platforms they used, and whether the information caused reassurance, confusion, or concern. Nurses can also support digital health literacy by guiding women to reliable websites, evidence-based mobile applications, and institutional or professional resources; explaining how to identify commercial bias, unsupported claims, and misinformation; and encouraging women to discuss online information with healthcare professionals before making decisions about symptom management. Such strategies may support informed decisionmaking and strengthen continuity of menopause care between clinical visits.

The participants’ mean MAAS total score was 53.04 ± 10.36. Since this mean is above the scale midpoint, it indicates that the women held a positive attitude toward menopause. Similarly, positive attitudes toward menopause have also been reported among female health professionals, with a mean MAAS total score of 46.15 ± 11.68^(17)^. In contrast, some studies have reported negative attitudes toward menopause^(12,29,30)^.

In our study, factors that may have contributed to women’s positive attitudes include the low prevalence of chronic disease, economic security, higher educational attainment, lower severity of menopausal symptoms, and the fact that women did not necessarily trust every piece of information obtained online/social media about menopausal symptoms and that such information did not affect their attitudes toward menopause. However, this interpretation should be made cautiously because the sample had relatively high educational attainment and a large proportion of participants reported income equal to expenses. Women with lower socioeconomic status, lower educational levels, limited access to digital technologies, or lower digital health literacy may have different attitudes toward menopause and different patterns of online information-seeking. Therefore, the positive attitude observed in this sample may not fully reflect the experiences of socioeconomically disadvantaged women or women with limited internet access.

The mean MRS total score among participants was 16.95 ± 6.67, indicating a noticeable symptom burden in the sample, although direct comparisons should be made cautiously because symptom levels may vary according to sample characteristics, menopausal status, and eligibility criteria. In this study, the observed symptom levels may be partly related to the participants’ higher education levels, positive attitudes toward menopause, and the exclusion of women using psychiatric or menopause-related medications. In other studies, reported mean MRS total scores have ranged from 7.53 ± 6.47 to 22.67 ± 8.06^(13,17)^ thus, the scores observed in our sample are broadly comparable to the existing literature.

### Limitations

This study has several limitations that should be considered when interpreting the findings. First, the analytical crosssectional design precludes causal inferences; the directionality between internet/social media use, attitudes toward menopause, and symptom severity cannot be established. It is possible that women with more severe symptoms were more likely to seek online information, rather than online information-seeking directly influencing symptom severity. Secondly, participants were recruited using a non-probability convenience approach from gynecology and family medicine units of a single university hospital in Çanakkale province. Therefore, selection bias is possible, and the findings may not be generalizable to communitydwelling women, women who do not seek health services, or women receiving care in different healthcare settings. Thirdly, the exclusion of women with diagnosed psychiatric disorders, those using psychiatric medication, and those using medication for menopausal symptoms may have reduced the inclusion of women with more severe psychological or menopausal symptoms. As a result, symptom severity in the sample may have been underestimated. Fourth, the study relied on self-reported measures, which may be subject to recall and social desirability bias; although questionnaires were completed individually, the supervised setting may have influenced responses. Fifth, internet/social media use was assessed using researcher-developed self-report items, and digital health literacy was not measured directly. Finally, the study did not objectively assess the quality, source, or type of menopause-related online content accessed by participants. Future longitudinal, multi-centre, and communitybased studies with more detailed assessment of digital health literacy and online content quality are warranted.

### Relevance for Clinical Practice

Based on the findings of this study, women’s health nurses should routinely assess climacteric women’s menopause-related online information-seeking during consultations and follow-up care. Practical strategies may include asking women whether they search for menopause-related information online before seeking care, discussing the sources they use, providing curated lists of reliable websites and evidence-based mobile applications, and teaching brief criteria for evaluating digital content, such as authorship, institutional credibility, date of update, references to scientific evidence, and the presence of commercial bias. Nurses can also encourage women to discuss online information before making decisions about symptom management. Integrating evidence-based eHealth literacy training into undergraduate nursing curricula and continuing professional development may strengthen nurses’ capacity to address misinformation, support informed decision-making, and promote timely professional help-seeking during the menopausal transition.

## CONCLUSION

In this study, women’s attitudes toward menopause were not significantly associated with their internet/social media use patterns. However, obtaining information about menopause online and daily internet/social media use were positively and significantly associated with menopausal symptom severity. A moderate general internet/social media use pattern was associated with lower symptom severity; however, this finding should not be interpreted as evidence of a protective effect or as menopause-specific engagement because the variable reflected general internet/social media use frequency and duration. These findings underscore the importance of integrating digital health literacy into menopause counselling. Women’s health nurses and other healthcare professionals should proactively inquire about online information-seeking, guiding women toward reliable resources, and supporting informed decision-making during the climacteric period.

## Data Availability

The data supporting the findings of this study are available from the corresponding author upon reasonable request.

## SUPPLEMENTARY MATERIAL

The following online material is available for this article
