# Supplementary material for: Menopausal symptoms, attitudes toward menopause, and social media/internet use among women in the climacteric period: a cross-sectional study
Source: Rev Esc Enferm USP. 2026 Aug 14;60:e20260107. doi: 10.1590/1980-220X-REEUSP-2026-0107en (PMC13475798; doi:10.1590/1980-220X-REEUSP-2026-0107en)
Supplement: Translation

# Translation: Síntomas de la menopausia, actitudes hacia la menopausia y uso de las redes sociales e Internet entre mujeres en el período climatérico: un estudio transversal

**DOI:** 10.1590/1980-220X-REEUSP-2026-0107es

**Authors:** Nazlı Emel Özer Yurdal, Emine Gerçek Öter, İbrahim Eren Pek, Mehmet Nuri Duran

**Affiliations:** 1 Çanakkale Onsekiz Mart University, Research Hospital, Obstetrics and Gynaecology Nursing, Çanakkale, Turkey.; 2 Aydın Adnan Menderes University, Faculty of Nursing, Department of Obstetrics and Gynecology Nursing, Aydın, Turkey.; 3 Çanakkale Onsekiz Mart University, Faculty of Medicine, Obstetrics and Gynaecology, Çanakkale, Turkey.; 4 Ministry of Health Ezine State Hospital, Department of Obstetrics and Gynaecology, Çanakkale, Turkey.

**Keywords:** Climaterio, Alfabetización en Salud, Internet, Menopausia, Enfermería, Medios de Comunicación Sociales

## Abstract

**Objetivo:**

Examinar las asociaciones entre la gravedad de los síntomas de la menopausia, las actitudes hacia la menopausia y el uso de Internet y las redes sociales entre las mujeres en el período climatérico.

**Método:**

Este estudio analítico transversal incluyó a 250 mujeres de entre 40 y 64 años que acudían a las unidades de hospitalización de ginecología y a las clínicas ambulatorias de medicina familiar de un hospital universitario. Los datos se recolectaron entre julio y septiembre de 2025, tras la aprobación del comité de ética. Las participantes completaron cuestionarios que incluían escalas validadas para evaluar las actitudes hacia la menopausia y la gravedad de los síntomas.

**Resultados:**

La mayoría de las participantes se encontraban en la posmenopausia (61,2 %); la edad promedio fue de 49,2 años. Los indicadores de uso de Internet y redes sociales no se asociaron con las actitudes hacia la menopausia. Una mayor gravedad de los síntomas se asoció con un mayor tiempo diario de uso de Internet y redes sociales, la búsqueda de información relacionada con la menopausia en línea y la búsqueda de información en línea antes de la consulta médica. Un patrón moderado de uso general de Internet y las redes sociales (2 a 3 veces por semana y de 1 a 3 horas al día) se asoció con una menor gravedad de los síntomas.

**Conclusión:**

La búsqueda de información en línea se asoció con la gravedad de los síntomas de la menopausia, pero no con las actitudes. Las enfermeras y otros profesionales de la salud deben evaluar la búsqueda de información en línea y fomentar la alfabetización digital en salud, orientando a las mujeres hacia recursos confiables.

## INTRODUCCIÓN

La menopausia es una etapa natural de la vida y se define clínicamente como el cese permanente de la menstruación, diagnosticado de manera retrospectiva tras 12 meses consecutivos de amenorrea en ausencia de otras causas patológicas o fisiológicas^(1)^. El período climatérico es más amplio que la menopausia e incluye los últimos años de la edad reproductiva, la transición a la menopausia y la posmenopausia temprana. Aunque el sistema de estadificación STRAW+10 proporciona un marco estandarizado para el envejecimiento reproductivo, el presente estudio lo considera únicamente como un marco conceptual para definir el período climatérico y no lo utiliza como criterio analítico de estadificación^(2)^.

La transición a la menopausia suele ir acompañada de síntomas vasomotores, trastornos del sueño, problemas de estado de ánimo y cognitivos, y síntomas genitourinarios, los cuales pueden afectar sustancialmente la calidad de vida y el funcionamiento diario^(1,3)^. La terapia hormonal sigue siendo la opción más eficaz para controlar los síntomas vasomotores y el síndrome genitourinario de la menopausia cuando no existe contraindicación, y las decisiones de tratamiento deben ser individualizadas^(3)^. Más allá de los cambios biológicos, las actitudes y expectativas de las mujeres hacia la menopausia desempeñan un papel importante en la forma en que se perciben y reportan los síntomas; la evidencia sugiere que las actitudes más negativas suelen estar asociadas con un mayor reporte de síntomas, mientras que las actitudes más positivas pueden amortiguar la experiencia de los síntomas^(4)^.

Al mismo tiempo, Internet y las redes sociales se han convertido en canales importantes a través de los cuales las mujeres buscan información de salud. Las redes sociales se definen comúnmente como un conjunto de aplicaciones basadas en Internet que se apoyan en los fundamentos ideológicos y tecnológicos de la Web 2.0 y permiten la creación y el intercambio de contenido generado por los usuarios^(5)^. Los estudios muestran que muchas mujeres menopáusicas buscan activamente en línea información sobre los síntomas, las opciones de manejo y las experiencias de otras mujeres^(6)^. Sin embargo, el entorno en línea también facilita la rápida difusión de contenido incompleto, engañoso o con sesgo comercial — una «infodemia»— que puede dificultar que las personas identifiquen orientación confiable y puede influir en las creencias sobre la salud y los comportamientos de búsqueda de cuidados de salud^(7)^.

Turquía está atravesando un proceso de envejecimiento demográfico; según los resultados del Sistema de Registro de Población por Domicilio de 2024, la edad media en Turquía aumentó a 34,4 años^(8)^. La provincia de Çanakkale se encuentra entre las regiones con una estructura de edad más avanzada, con una edad media de 41,7 años en 2024 (40,8 para los hombres y 42,6 para las mujeres) ^(9)^. Este perfil se aproxima a la edad media reportada para la Unión Europea (44,7 años al 1 de enero de 2024) ^(10)^. Por lo tanto, estudiar las experiencias relacionadas con la menopausia en la provincia de Çanakkale puede aportar conocimientos relevantes para entornos con estructuras demográficas comparativamente más envejecidas.

Dado el papel cada vez más importante de las fuentes de información digital, el asesoramiento sobre la menopausia también debería incorporar el apoyo a la alfabetización digital en salud (alfabetización en eSalud), que se refiere a las habilidades percibidas por las personas para encontrar, evaluar y aplicar información de salud electrónica a los problemas de salud^(11)^. En este contexto, este estudio transversal tiene como objetivo examinar si los patrones de uso de Internet y las redes sociales — en particular, la búsqueda en línea de información relacionada con la menopausia— se asociaban con la gravedad de los síntomas menopáusicos y las actitudes hacia la menopausia entre las mujeres en el período climatérico. Específicamente, el estudio tiene como objetivo (1) describir el nivel de gravedad de los síntomas de la menopausia; (2) describir las actitudes de las mujeres hacia la menopausia; (3) evaluar las asociaciones entre el uso de Internet y las redes sociales, por un lado, y tanto la gravedad de los síntomas como las actitudes, por otro; y (4) caracterizar los patrones generales de uso de Internet y las redes sociales por parte de las mujeres, así como sus comportamientos de búsqueda de información en línea relacionada con la menopausia.

## MÉTODOS

### Diseño del estudio

El diseño utilizado es una metodología analítica transversal

### Consideraciones Éticas

Se obtuvo el consentimiento informado por escrito para participar en el estudio de todas las participantes, de conformidad con la Declaración de Helsinki. Se obtuvo la aprobación del Comité de Ética en Investigación Clínica de la Universidad Çanakkale Onsekiz Mart mediante la resolución 2025-07/0702 y el protocolo n.º 2025/97, con fecha del 17 de abril de 2025. Se obtuvo el permiso institucional de las unidades hospitalarias pertinentes antes del proceso de recolección de datos. Posteriormente, se obtuvo el permiso por escrito para recolectar datos de la Oficina del Médico Jefe del Hospital Universitario Çanakkale Onsekiz Mart.

### Entorno y participantes

El estudio se llevó a cabo en la unidad de hospitalización de ginecología y en las clínicas ambulatorias de medicina familiar del Hospital Universitario Çanakkale Onsekiz Mart. Los datos se recolectaron entre julio de 2025 y septiembre de 2025, tras haber obtenido la aprobación del comité de ética.

### Tamaño de la Muestra

Según los registros clínicos, aproximadamente 1 500 mujeres de 40 años o más acuden a estas clínicas cada año. Se realizó un cálculo a priori del tamaño de la muestra utilizando G*Power 3.1. Suponiendo un tamaño de efecto medio (d de Cohen = 0,50), un nivel alfa de 0,05 y una potencia del 95 % (1–β = 0,95), el tamaño mínimo de muestra requerido fue de 202 participantes. Para compensar la posible falta de respuesta y los datos incompletos, el tamaño de muestra objetivo se fijó en 250 mujeres.

### Criterios de Elegibilidad

#### Criterios de inclusión

- edad entre 40 y 64 años;
- disposición a participar y firma del consentimiento informado;
- ausencia de trastornos psiquiátricos diagnosticados;
- no estar tomando medicamentos psiquiátricos; y
- no estar tomando ningún medicamento para los síntomas de la menopausia.

#### Criterios de exclusión

- retirada del consentimiento o falta de disposición para continuar participando; y
- cuestionarios incompletos o problemas técnicos durante la recolección de datos.

### Herramientas Para la Recolección de Datos

Los datos del estudio se recolectaron mediante un cuestionario de tres partes: (1) un formulario de información personal que indagaba las características sociodemográficas y biográficas de las mujeres, así como sus antecedentes relacionados con la menopausia; (2) la Escala de Evaluación de Actitudes hacia la Menopausia (Menopause Attitude Assessment Scale - MAAS, por sus siglas en inglés; versión en turco); y (3) la Escala de Calificación de la Menopausia (Menopause Rating Scale - MRS, por sus siglas en inglés; versión en turco)^(12,13,14,15,16,17,18)^.

### Cuestionario

El cuestionario fue elaborado por los investigadores, siguiendo la literatura^(13,14,15,16,17,18,19,20,21,22)^. Constaba de 26 ítems diseñados para recabar las características socioculturales y demográficas de las mujeres (p. ej., edad, nivel educativo, situación económica), las características relacionadas con la menopausia y las características del uso de medios digitales y redes sociales (p. ej., uso diario de redes sociales e Internet, uso de Internet o redes sociales para obtener información relacionada con la menopausia y búsquedas en línea antes de consultar a un médico).

El ítem sobre el uso semanal de Internet y las redes sociales evaluaba el uso general de las redes sociales e Internet por parte de las participantes, no el uso de contenido específico sobre la menopausia. Este ítem incluía tres categorías de frecuencia (una vez por semana, 2–3 veces por semana y 6–7 días por semana) y pedía a las participantes que informaran su duración diaria aproximada de uso. Para el análisis, la duración diaria reportada se clasificó en <1 hora, 1–3 horas y ≥4 horas. Por lo tanto, la categoría «2–3 veces por semana y 1–3 horas por día» se construyó combinando la categoría de frecuencia semanal «2–3 veces por semana» con la categoría de duración diaria «1–3 horas por día». Esta variable se interpretó como un patrón moderado de uso general de Internet y las redes sociales, y no como una participación específica relacionada con la menopausia ni como una exposición protectora.

El borrador del cuestionario (que incluía el Formulario de Información Personal y los ítems sobre el uso de las redes sociales e Internet), junto con las instrucciones para la administración de la escala, se envió a 20 expertos para evaluar la validez de contenido y la claridad. Se planeó que el panel de expertos incluyera a profesores y enfermeras clínicas en el área de Enfermería de Salud de la Mujer y Obstetricia, especialistas en Obstetricia y Ginecología, Medicina Familiar y Salud Pública, y un experto en medición, evaluación y psicometría. Cada ítem se calificó en una escala de tres puntos: «No apropiado (0)» / «Apropiado, pero necesita revisión (1)» / «Apropiado (2)». De los 20 expertos invitados, 12 respondieron; el alfa de Krippendorff = 0,833 indicó un alto nivel de concordancia entre los expertos. Se realizaron revisiones de lenguaje y contenido basadas en los comentarios de los expertos, y se elaboró la versión final del formulario. Este formulario se proporciona como archivo complementario 1.

*Prueba piloto del cuestionario desarrollado:* Se realizó una prueba piloto con una versión casi definitiva entre 8 mujeres menopáusicas seleccionadas de la población objetivo. La prueba piloto demostró que los ítems eran comprensibles, las instrucciones adecuadas y el tiempo de completación factible; por lo tanto, se procedió a la recolección completa de datos. Los datos de la prueba piloto no se incluyeron en el análisis final.

### Escala de Evaluación de Actitudes hacia la Menopausia (MAAS)

Desarrollada originalmente en 1994 para medir las actitudes hacia la menopausia y el período posmenopáusico en diferentes grupos de edad en Turquía, la escala contiene 2 enunciados positivos y 18 negativos. Los ítems positivos se califican de 0 («totalmente en desacuerdo») a 4 («totalmente de acuerdo»); los ítems negativos se califican a la inversa. Las puntuaciones totales posibles oscilan entre 0 y 80; las puntuaciones más altas indican una actitud más positiva hacia la menopausia, mientras que las más bajas indican una actitud negativa. Las puntuaciones por encima de la media reflejan actitudes cada vez más positivas^(14)^. Se informó que la confiabilidad de la escala fue de 0,86^(15)^. Este formulario se proporciona como archivo complementario 2.

### Escala de Evaluación de la Menopausia (MRS — versión en turco):

La MRS, desarrollada originalmente en 1992 y adaptada al turco en 2005, consta de 11 ítems, tres subescalas y un formato de Likert de 5 puntos (ninguno, leve, moderado, grave, muy grave). Las puntuaciones totales oscilan entre 0 y 44. Las tres subescalas son: Somática (ítems 1, 2, 3, 11), Psicológica (ítems 4, 5, 6, 7) y Urogenital (ítems 8, 9, 10). Un aumento en la puntuación total refleja un aumento en la gravedad de los síntomas y un deterioro en la calidad de vida (16, 19). El coeficiente de confiabilidad alfa de Cronbach de la versión turca es de 0,84^(16,19)^. Esta versión se proporciona como archivo complementario 3.

### Recolección de Datos

Los datos se recolectaron entre julio de 2025 y septiembre de 2025 en la unidad de hospitalización de ginecología y en las clínicas ambulatorias de medicina familiar del Hospital Universitario Onsekiz Mart de Çanakkale. En los días en que los investigadores estaban disponibles (incluidos los días laborables, fuera del horario laboral y los fines de semana), se abordó de manera consecutiva a las mujeres elegibles y se les informó sobre el propósito del estudio, los procedimientos, la confidencialidad y su derecho a negarse a participar o a retirarse en cualquier momento sin consecuencias. Se obtuvo el consentimiento informado por escrito antes de la participación.

Las participantes completaron los cuestionarios utilizando un formato autoadministrado (autoinforme) en un área adecuada que permitía la privacidad. Un investigador capacitado permaneció disponible durante la cumplimentación para aclarar dudas sobre el procedimiento (por ejemplo, cómo marcar las respuestas) y para garantizar que los formularios se completaran de manera individual, pero no orientó ni influyó en las respuestas de las participantes. La cumplimentación tomó aproximadamente de 15 a 20 minutos. Tras su entrega, los cuestionarios se revisaron en el lugar para detectar respuestas faltantes; si algún ítem se había dejado en blanco sin intención, se preguntaba a los participantes si deseaban completarlo. Se excluyeron los formularios con datos faltantes sustanciales o afectados por problemas técnicos o administrativos, de acuerdo con los criterios de exclusión. El reclutamiento continuó hasta alcanzar el tamaño de muestra objetivo.

### Análisis de Datos

Los datos se analizaron utilizando el paquete estadístico JAMOVI (versión 2.6.44-solid). La normalidad se evaluó con las pruebas de Kolmogorov–Smirnov y Shapiro–Wilk. Las estadísticas descriptivas se presentaron como recuento, porcentaje, media, desviación estándar y valores mínimo–máximo. Las relaciones entre las puntuaciones totales de las escalas se examinaron mediante la correlación de rangos de Spearman (ρ).

### Directrices de Presentación de Informes

Este estudio transversal se presentó de acuerdo con la declaración STROBE; la lista de verificación completada se proporciona como archivo complementario 4.

## RESULTADOS

Se analizó a un total de 250 mujeres. La Tabla 1 presenta las características demográficas recogidas en el cuestionario elaborado por los investigadores (sección demográfica), incluyendo la edad (categorizada; media general ± DE 49,2 ± 6,50), el nivel educativo, la situación laboral, el estado civil y la situación económica, el número de partos, las enfermedades crónicas y el consumo de tabaco y alcohol.

**Tabla 1:** Distribución de las características descriptivas de las mujeres (N = 250 – provincia de Çanakkale, Turquía, 2025.

Variable	n	%
Edad (años)^*^		
40–43	59	23,6
44–48	74	29,6
49–53	47	18,8
54–64	70	28,0
**Media ± DE = 49,2 ± 6,50**		
Nivel educativo		
Bachillerato	69	27,6
Escuela de formación profesional (programa de dos años con título de técnico superior)	25	10,0
Grado universitario	128	51,2
Posgrado	28	11,2
**Empleo en un trabajo que genera ingresos**		
Sí	192	76,8
No	58	23,2
**Estado civil**		
Casado	194	77,6
Soltero	45	18,0
Otros	11	4,4
**Situación económica**		
Los ingresos igualan a los gastos	149	59,6
Los ingresos son inferiores a los gastos	77	30,8
Ingresos superiores a los gastos	24	9,6
**Hijos**		
Sí	183	73,2
No	67	26,8
**Enfermedad crónica**		
Presente	79	31,6
Ausente	171	68,4
**Tabaquismo**		
Sí	92	36,8
No	158	63,2
**Consumo de alcohol**		
Sí	42	16,8
No	208	83,2

*Los datos se obtuvieron a partir de un cuestionario elaborado por los investigadores (instrumento de 26 ítems).

Los valores se muestran como n (%) salvo que se indique lo contrario; la edad se presenta en categorías y también se proporciona la media global ± DE.

El 29,6 % de las mujeres se encontraba en el grupo de edad de 44 a 48 años; la edad media era de 49,2 ± 6,50 años. En general, el 51,2 % poseía una licenciatura, el 76,8 % tenía un empleo remunerado, el 77,6 % estaba casada y el 59,6 % declaró que sus ingresos igualaban a sus gastos. Además, el 73,2 % tenía hijos, el 68,4 % declaró no padecer ninguna enfermedad crónica, el 63,2 % no fumaba y el 83,2 % declaró no consumir alcohol (Tabla 1).

Entre las participantes, el 61,2 % había entrado en la menopausia. Entre estas mujeres, el tiempo medio transcurrido desde la menopausia era de 3,61 ± 3,98 años. Cuando se les preguntó por sus sentimientos en el momento de entrar en la menopausia, el 26,4 % declaró haber experimentado emociones negativas (p. ej., tristeza, sensación de pérdida) (Tabla 2).

**Tabla 2:** Características relacionadas con la menopausia de las participantes (N = 250) – provincia de Çanakkale, Turquía, 2025.

Característica	n	%
¿**Estás en la menopausia?**		
Sí	153	61,2
No	97	38,8
Tiempo transcurrido desde el inicio de la menopausia (años)^*^	**Media**	**DE**
	3,61	3,98
Sentimientos en el momento de entrar en la menopausia^*^		
Sentimientos negativos (tristeza, sensación de pérdida, etc.)	66	26,4
Sin sentimientos concretos	47	18,8
Sentimientos positivos (alivio, alegría, etc.)	40	16,0

* Respondieron únicamente las mujeres que ya habían entrado en la menopausia.

Los valores se expresan en n (%) salvo que se indique lo contrario; las variables continuas se presentan como media y desviación estándar.

Entre las mujeres del estudio, el 88,4 % declaró utilizar las redes sociales e Internet en su vida cotidiana. La proporción de las que utilizaban estos canales para buscar información sobre la menopausia fue del 55,6 %; de ellas, el 12,4 % consideraba que la información era fiable y el 15,2 % la encontraba útil. En total, el 88,0 % afirmó que la información obtenida en línea no influía en su actitud hacia la menopausia. Si bien el 51,6 % buscó en Internet o en las redes sociales formas de afrontar los síntomas de la menopausia, el 84,8 % no consideró útil esta información; además, el 51,6 % declaró haber buscado información en línea antes de consultar a un médico. En cuanto a los patrones de uso, el 79,2 % utilizaba plataformas en línea al menos una vez a la semana, y el 83,2 % pasaba entre 1 y 3 horas al día en Internet. La plataforma más utilizada fue el motor de búsqueda de Google (32,4 %), y el dispositivo de acceso más habitual fue el teléfono móvil (69,6 %). Por último, el 33,2 % de las mujeres afirmó que le gustaría recibir información sobre los síntomas de la menopausia y cómo afrontarlos de enfermeras, comadronas o profesionales sanitarios, y el 28,8 % de los médicos de familia (Tabla 3).

**Tabla 3:** SUso de las redes sociales e Internet entre las participantes (N = 250) – provincia de Çanakkale, Turquía, 2025.

Pregunta	n	%
**¿Utiliza las redes sociales e Internet en su vida cotidiana?**		
Sí	221	88,4
No	29	11,6
**¿Utilizas las redes sociales o Internet para informarte sobre la menopausia?**		
Sí	139	55,6
No	111	44,4
**¿Confías en la información que encuentras en Internet sobre la menopausia?**		
Sí	31	12,4
No	219	87,6
**¿Te resulta útil la información que encuentras en Internet sobre los síntomas de la menopausia?**		
Sí	38	15,2
No	212	84,8
**¿Crees que lo que has aprendido en Internet influye en tu actitud hacia la menopausia?**		
Sí	30	12,0
No	220	88,0
Dirección del efecto sobre la actitud (n = 30)^*^		
Positiva	5	16,7
Negativa	25	83,3
**¿Ha buscado información en Internet sobre cómo afrontar los síntomas de la menopausia?**		
Sí	129	51,6
No	121	48,4
**¿Te resulta útil la información que has obtenido en Internet sobre cómo afrontar los síntomas?**		
Útil	38	15,2
No útil	212	84,8
**¿Buscas información en Internet antes de acudir al médico por síntomas de la menopausia?**		
Sí	129	51,6
No	121	48,4
**Frecuencia de uso de las redes sociales e Internet**		
Una vez a la semana	198	79,2
2-3 veces por semana	48	19,2
6-7 días a la semana	4	1,6
**Tiempo diario dedicado a Internet**		
< 1 hora	22	8,8
1-3 horas	208	83,2
4-7 horas y más	20	8,0
Plataformas más utilizadas^*^		
Facebook	31	12,4
X (antiguo Twitter)	9	3,6
Instagram	77	30,8
YouTube	48	19,2
Google (motor de búsqueda)	81	32,4
TikTok	4	1,6
Dispositivos utilizados para las redes sociales^*^		
Teléfono móvil	174	69,6
Ordenador	56	22,4
Tableta	20	8,0
**Fuentes de información sobre los síntomas de la menopausia y cómo afrontarlos**		
Colegio/educación	40	16,0
Médico de familia	72	28,8
Enfermera/comadrona/profesional sanitario	83	33,2
Hospital	30	12,0
Amigos	8	3,2
Libros/revistas/tesis/revistas electrónicas	1	0,4
Fuentes de Internet	6	2,4
Aplicaciones móviles sobre la menopausia	4	1,6
Televisión	6	2,4

*Se permiten respuestas múltiples.

La puntuación total media de las mujeres en la escala MRS (versión turca) fue de 16,95 ± 6,67. Por subescalas, las puntuaciones medias fueron de 5,98 ± 2,82 para los síntomas psicológicos, 6,76 ± 2,61 para los somáticos y 4,20 ± 2,05 para los urogenitales (Tabla 4). Las puntuaciones mínima y máxima de la MAAS (versión turca) fueron de 26 y 80, respectivamente; la puntuación media total de la MAAS fue de 53,04 ± 10,36 (Tabla 4).

**Tabla 4:** Puntuaciones en la Escala de Valoración de la Menopausia (MRS — versión turca) y en la Escala de Evaluación de la Actitud ante la Menopausia (MAAS — versión turca) (N = 250) - provincia de Çanakkale, Turquía, 2025.

Escala / subescala	Rango posible	Mín. y máx. observados	Media ± DE
Total de la MAAS	0–80	26–80	**53,04 ± 10,36**
MRS total	0–44	3–43	**16,95 ± 6,67**
Subescalas de la MRS			
Síntomas psicológicos	0–15	0–15	**5,98 ± 2,82**
Síntomas somáticos	0–16	0–16	**6,76 ± 2,61**
Síntomas urogenitales	0–13	0–13	**4,20 ± 2,05**

* *Nota.* Las puntuaciones más altas en la escala MRS indican una mayor gravedad de los síntomas; las puntuaciones más altas en la escala MAAS indican una actitud más positiva hacia la menopausia. Los valores se presentan como mínimo y máximo observados y media ± DE.

Al analizar la relación entre el uso de las redes sociales e Internet por parte de las mujeres y la puntuación total del MAAS, no se encontraron asociaciones significativas en cuanto al uso diario de las redes sociales e Internet, el uso de estos canales para buscar información relacionada con la menopausia, la confianza en la información obtenida en línea o la percepción de que dicha información resulta útil. Por el contrario, la obtención de información de Internet o las redes sociales para hacer frente a los síntomas de la menopausia mostró una relación negativa y no significativa con la puntuación total del MAAS; la búsqueda de información relacionada con la menopausia y la confianza en la información obtenida en línea también mostraron relaciones no significativas con la puntuación total del MAAS. Además, la búsqueda en Internet o en las redes sociales antes de acudir al médico por síntomas de la menopausia y tener un patrón moderado de uso general de Internet o las redes sociales —definido como el uso de las redes sociales o de Internet entre 2 y 3 veces por semana y entre 1 y 3 horas al día— mostraron asociaciones positivas, aunque no significativas, con la puntuación total del MAAS (Tabla 5).

**Tabla 5:** Correlaciones de Spearman entre el uso de las redes sociales e Internet y las puntuaciones totales del MAAS y el MRS (N = 250) - provincia de Çanakkale, Turquía, 2025.

Variables	Total del MAAS Media		Total del MRS Media	
	r	p	r	p
Uso de las redes sociales e Internet en la vida cotidiana	0,058	0,363	0,523	<0,001
Uso de las redes sociales o de Internet para obtener información sobre la menopausia	0,002	0,975	0,691	<0,001
Confiar en la información obtenida en Internet sobre la menopausia	–0,024	0,711	0,009	0,893
Considerar útil la información en línea sobre los síntomas de la menopausia	0,031	0,628	–0,110	0,083
Buscar información en Internet sobre cómo afrontar los síntomas de la menopausia	–0,044	0,488	–0,117	0,065
Buscar información en Internet antes de acudir al médico por síntomas de la menopausia	0,092	0,147	0,644	<0,001
Patrón moderado de uso de Internet y redes sociales: de 2 a 3 veces por semana y de 1 a 3 horas al día	0,059	0,354	–0,237	<0,001

*Nota. Las correlaciones son el coeficiente ρ de Spearman. MAAS = Escala de Evaluación de Actitudes ante la Menopausia (versión turca); MRS = Escala de Valoración de la Menopausia (versión turca). Se considera estadísticamente significativo un valor de p < 0,05. Las correlaciones son el coeficiente ρ de Spearman con los valores p correspondientes.

Según el análisis de correlación de Spearman, el uso diario de las redes sociales e Internet por parte de las mujeres, el uso de estas plataformas para buscar información relacionada con la menopausia y la búsqueda en línea antes de consultar a un médico por síntomas de la menopausia se asociaron de manera positiva y significativa con el puntaje total del MRS. Por el contrario, el patrón moderado de uso general de Internet y las redes sociales —definido como el uso de estas plataformas de 2 a 3 veces por semana y de 1 a 3 horas al día— se asoció de manera negativa y significativa con la puntuación total del MRS. Esta variable refleja la frecuencia y la duración del uso general de Internet y las redes sociales, más que el uso de contenido específico sobre la menopausia. Considerar útil la información en línea sobre los síntomas de la menopausia y buscar información en línea sobre cómo lidiar con dichos síntomas mostraron correlaciones negativas, aunque no significativas, con la puntuación total del MRS. Confiar en la información en línea relacionada con la menopausia no se asoció de manera significativa con la puntuación total del MRS. Debido al diseño transversal, estos hallazgos deben interpretarse como asociaciones y no indican la dirección de la relación.

## DISCUSIÓN

En este estudio, examinamos si el uso de Internet y las redes sociales por parte de las mujeres se asociaba con las actitudes hacia la menopausia y los síntomas de la menopausia. En general, las actitudes hacia la menopausia no se asociaron de manera significativa con el uso diario de Internet o las redes sociales, el uso de estos canales con fines relacionados con la menopausia, la búsqueda en línea antes de consultar a un médico, la obtención de información en línea para sobrellevar los síntomas de la menopausia, la confianza en la información obtenida en línea o la percepción de que esta es útil. Por el contrario, la gravedad de los síntomas de la menopausia varió según comportamientos específicos en línea: el uso diario de Internet y las redes sociales, el uso de estos canales para obtener información relacionada con la menopausia y la búsqueda en línea antes de consultar a un médico por los síntomas de la menopausia se asociaron de manera positiva y significativa con el puntaje total del MRS, mientras que un patrón moderado de uso general de Internet y las redes sociales (2 a 3 veces por semana y de 1 a 3 horas al día) se asoció de manera negativa y significativa con el puntaje total del MRS. Dado que esta variable se derivó del ítem de uso general de Internet y las redes sociales, no debe interpretarse como una medida de la participación específica relacionada con la menopausia. Sin embargo, el diseño transversal no permite determinar si los síntomas más graves llevaron a las mujeres a buscar información en línea con mayor frecuencia, o si la exposición a contenido en línea relacionado con la menopausia influyó en la percepción de los síntomas. Por lo tanto, todos los hallazgos deben interpretarse como asociaciones y no como efectos causales.

La confianza en la información en línea sobre la menopausia y la percepción de que dicha información es útil no se asociaron con la gravedad de los síntomas. En conjunto, estos hallazgos sugieren que el propósito y el patrón de participación en línea pueden ser más importantes que el uso de Internet por sí solo, y resaltan la importancia clínica de evaluar de manera rutinaria la búsqueda de información en línea y de fomentar la alfabetización digital en salud, orientando a las mujeres hacia recursos confiables.

Brindar a las mujeres educación planificada sobre la menopausia contribuye a un desarrollo positivo en las actitudes hacia la menopausia^(23)^. También se ha informado que la mayoría de las mujeres había recibido previamente información sobre la menopausia, principalmente a través de la televisión, y que su calidad de vida se vio afectada negativamente^(24)^. Las actitudes de las mujeres hacia la menopausia pueden variar; algunas expresan actitudes positivas y otras, negativas. Además, es posible que una proporción considerable de mujeres no cuente con conocimientos suficientes sobre la menopausia y que utilice principalmente las redes sociales e Internet para obtener información relacionada con la menopausia. El contenido de los medios de comunicación a veces puede enfatizar los aspectos negativos de la menopausia, mientras que destaca los aspectos positivos con menos frecuencia, lo que puede aumentar la ansiedad y las preocupaciones de las mujeres respecto a la menopausia. Por lo tanto, la menopausia no debe plantearse como un problema excesivamente médico^(25)^. Uno de los hallazgos notables de este estudio fue la aparente discrepancia entre los altos niveles de búsqueda de información digital y los bajos niveles de confianza y utilidad percibida de la información en línea relacionada con la menopausia. Aunque más de la mitad de las participantes informaron que utilizaban Internet o las redes sociales para buscar información sobre la menopausia y que realizaban búsquedas en línea antes de consultar a un médico, solo una pequeña pro-porción declaró confiar en esta información o encontrarla útil. Este patrón puede indicar que las mujeres tienen necesidades de información no satisfechas durante el período climatérico, pero también pueden experimentar dificultades para evaluar la credibilidad, la precisión y la aplicabilidad del contenido en línea relacionado con la menopausia. Para la práctica de enfermería, este hallazgo sugiere que preguntar a las mujeres qué han buscado en línea, qué fuentes han utilizado y si confían en ellas puede ser un componente importante del asesoramiento sobre la menopausia. Este enfoque puede ayudar a las enfermeras a identificar la desinformación, corregir malentendidos y orientar a las mujeres hacia recursos basados en la evidencia.

El uso diario de Internet y las redes sociales por parte de las mujeres, su uso de estos canales para obtener información relacionada con la menopausia y la búsqueda en línea antes de consultar a un médico se asociaron de manera positiva y significativa con la gravedad de los síntomas de la menopausia. Sin embargo, no se puede determinar la dirección de estas asociaciones. Las mujeres que experimentan síntomas más graves pueden ser más propensas a buscar información en línea antes de buscar atención profesional. Por el contrario, la exposición repetida a información incompleta, inconsistente o que provoca ansiedad puede influir en la forma en que se perciben los síntomas. El hallazgo de que un patrón moderado de uso general de Internet y las redes sociales se asoció con una menor gravedad de los síntomas también debe interpretarse con cautela. Esta variable refleja la frecuencia y la duración del uso general de Internet y las redes sociales, más que el uso de contenido específico sobre la menopausia. Por lo tanto, en lugar de indicar un efecto protector, esta asociación podría reflejar diferencias en el comportamiento digital general, la búsqueda selectiva de información u otros factores no medidos, como la educación, la alfabetización en salud o el acceso a información confiable.

En general, el uso de Internet y las redes sociales no mostró una asociación significativa con las actitudes hacia la menopausia en nuestra muestra, mientras que la gravedad de los síntomas varió según los comportamientos de búsqueda de información en línea. Intervenciones recientes sobre la menopausia basadas en dispositivos móviles e Internet también reportan reducciones en los síntomas vasomotores y conductuales entre las mujeres que interactúan con plataformas digitales^(19,20,21)^. En este sentido, nuestros hallazgos concuerdan con la literatura. Entre las mujeres de 40 años o más, no se encontró ninguna relación entre confiar en la información relacionada con la menopausia obtenida de Internet o las redes sociales, o considerarla útil, y los síntomas de la menopausia.

Es posible que algunas mujeres en el período climatérico no confíen en la información obtenida en línea o no la consideren útil; en consecuencia, se ha informado que solo una proporción limitada utiliza Internet para lidiar con los síntomas de la menopausia^(13)^. Se ha demostrado que la terapia cognitivo-conductual basada en Internet reduce los sofocos y mejora la calidad del sueño^(26)^. Entre las mujeres posmenopáusicas y de edad avanzada, el uso de las tecnologías de la información y la comunicación también se ha asociado con menores niveles de fragilidad^(22)^. Además, se ha informado que un programa digital de atención y mejora ha sido eficaz para ayudar a las mujeres a sobrellevar los síntomas de la menopausia^(19)^. Se ha encontrado una asociación significativa entre la alfabetización en salud y los síntomas de la menopausia, y la mejora de la alfabetización en salud en las mujeres menopáusicas se ha asociado con una mejor calidad de vida^(27)^. También se ha informado que muchas mujeres no reconocen los síntomas de la menopausia, necesitan apoyo o ayuda profesional, y podrían beneficiarse de plataformas desarrolladas para estas necesidades. Asimismo, se han identificado brechas en el conocimiento y la alfabetización sobre la menopausia; además, aproximadamente la mitad de las mujeres prefirió una intervención basada en la web, y la mayoría consideró útil una herramienta de e-salud basada en la terapia cognitivo-conductual y se mostró dispuesta a utilizarla^(28)^. Estos hallazgos tienen implicaciones prácticas para la atención de enfermería. Las enfermeras especializadas en salud de la mujer pueden incorporar preguntas breves sobre la búsqueda de información en línea en las consultas de rutina sobre la menopausia, como por ejemplo si las mujeres han buscado información relacionada con la menopausia en línea, qué sitios web o plataformas de redes sociales utilizaron, y si la información les generó tranquilidad, confusión o preocupación. Las enfermeras también pueden fomentar la alfabetización digital en salud al orientar a las mujeres hacia sitios web confiables, aplicaciones móviles basadas en evidencia y recursos institucionales o profesionales; al explicarles cómo identificar sesgos comerciales, afirmaciones sin fundamento y desinformación; y al alentarlas a discutir la información en línea con profesionales de la atención de salud antes de tomar decisiones sobre el manejo de los síntomas. Dichas estrategias pueden apoyar la toma de decisiones informadas y fortalecer la continuidad de la atención de la menopausia entre las visitas clínicas.

La puntuación total promedio de las participantes en la escala MAAS fue de 53,04 ± 10,36. Dado que esta media se encuentra por encima del punto medio de la escala, indica que las mujeres tenían una actitud positiva hacia la menopausia. De manera similar, también se han reportado actitudes positivas hacia la menopausia entre las profesionales de la salud, con una puntuación total promedio en la escala MAAS de 46,15 ± 11,68^(17)^. Por el contrario, algunos estudios han reportado actitudes negativas hacia la menopausia^(12,29,30)^.

En nuestro estudio, entre los factores que pueden haber contribuido a las actitudes positivas de las mujeres se incluyen la baja prevalencia de enfermedades crónicas, la seguridad económica, un mayor nivel educativo, una menor gravedad de los síntomas de la menopausia y el hecho de que las mujeres no necesariamente confiaban en toda la información obtenida en línea o en las redes sociales sobre los síntomas de la menopausia, y que dicha información no afectaba sus actitudes hacia la menopausia. Sin embargo, esta interpretación debe hacerse con cautela, ya que la muestra tenía un nivel educativo relativamente alto y una gran proporción de las participantes reportó ingresos iguales a los gastos. Las mujeres con un nivel socioeconómico más bajo, niveles educativos más bajos, acceso limitado a las tecnologías digitales o menor alfabetización digital en salud podrían tener actitudes diferentes hacia la menopausia y patrones distintos de búsqueda de información en línea. Por lo tanto, la actitud positiva observada en esta muestra podría no reflejar plenamente las experiencias de las mujeres en desventaja socioeconómica o de aquellas con acceso limitado a Internet.

La puntuación total promedio del MRS entre las participantes fue de 16,95 ± 6,67, lo que indica una carga de síntomas notable en la muestra, aunque las comparaciones directas deben realizarse con cautela, ya que los niveles de síntomas pueden variar según las características de la muestra, el estado menopáusico y los criterios de elegibilidad. En este estudio, los niveles de síntomas observados pueden estar relacionados en parte con los niveles de educación más altos de las participantes, sus actitudes positivas hacia la menopausia y la exclusión de mujeres que tomaban medicamentos psiquiátricos o relacionados con la menopausia. En otros estudios, las puntuaciones totales medias del MRS reportadas han oscilado entre 7,53 ± 6,47 y 22,67 ± 8,06^(13,17)^; por lo tanto, las puntuaciones observadas en nuestra muestra son, en términos generales, comparables a las de la literatura existente.

### Limitaciones

Este estudio presenta varias limitaciones que deben tenerse en cuenta al interpretar los resultados. En primer lugar, el diseño analítico transversal impide realizar inferencias causales; no se puede establecer la relación de causalidad entre el uso de Internet y las redes sociales, las actitudes hacia la menopausia y la gravedad de los síntomas. Es posible que las mujeres con síntomas más graves fueran más propensas a buscar información en línea, en lugar de que la búsqueda de información en línea influyera directamente en la gravedad de los síntomas. En segundo lugar, las participantes fueron reclutadas mediante un enfoque de conveniencia no probabilístico en las unidades de ginecología y medicina familiar de un único hospital universitario en la provincia de Çanakkale. Por lo tanto, es posible que exista un sesgo de selección, y es posible que los resultados no sean generalizables a las mujeres que viven en la comunidad, a las que no buscan servicios de salud o a las que reciben atención de salud en diferentes entornos. En tercer lugar, la exclusión de mujeres con trastornos psiquiátricos diagnosticados, aquellas que tomaban medicamentos psiquiátricos y las que tomaban medicamentos para los síntomas de la menopausia pudo haber reducido la inclusión de mujeres con síntomas psicológicos o de la menopausia más graves. Como resultado, es posible que se haya subestimado la gravedad de los síntomas en la muestra. En cuarto lugar, el estudio se basó en medidas autoinformadas, que pueden estar sujetas a sesgos de recuerdo y de deseabilidad social; aunque los cuestionarios se completaron de manera individual, el entorno supervisado pudo haber influido en las respuestas. En quinto lugar, el uso de Internet y las redes sociales se evaluó mediante ítems de autoinforme desarrollados por los investigadores, y la alfabetización en salud digital no se midió directamente. Por último, el estudio no evaluó objetivamente la calidad, la fuente o el tipo de contenido en línea relacionado con la menopausia al que accedieron las participantes. Se justifican futuros estudios longitudinales, multicéntricos y basados en la comunidad con una evaluación más detallada de la alfabetización en salud digital y la calidad del contenido en línea.

### Relevancia Para la Práctica Clínica

Con base en los hallazgos de este estudio, las enfermeras especializadas en salud de la mujer deben evaluar de manera rutinaria la búsqueda de información en línea relacionada con la menopausia que realizan las mujeres en la etapa climatérica durante las consultas y la atención de seguimiento. Las estrategias prácticas pueden incluir preguntar a las mujeres si buscan información relacionada con la menopausia en línea antes de acudir a recibir atención, analizar las fuentes que utilizan, proporcionar listas seleccionadas de sitios web confiables y aplicaciones móviles basadas en evidencia, y enseñar criterios breves para evaluar el contenido digital, tales como la autoría, la credibilidad institucional, la fecha de actualización, las referencias a evidencia científica y la presencia de sesgos comerciales. Las enfermeras también pueden alentar a las mujeres a hablar sobre la información en línea antes de tomar decisiones sobre el manejo de los síntomas. La integración de la capacitación en alfabetización digital en salud basada en la evidencia en los planes de estudio de pregrado de enfermería y en el desarrollo profesional continuo puede fortalecer la capacidad de las enfermeras para abordar la desinformación, apoyar la toma de decisiones informadas y promover la búsqueda oportuna de ayuda profesional durante la transición a la menopausia.

## CONCLUSIÓN

En este estudio, las actitudes de las mujeres hacia la menopausia no se asociaron significativamente con sus patrones de uso de Internet y las redes sociales. Sin embargo, la obtención de información sobre la menopausia en línea y el uso diario de Internet y las redes sociales se asociaron de manera positiva y significativa con la gravedad de los síntomas de la menopausia. Un patrón moderado de uso general de Internet y las redes sociales se asoció con una menor gravedad de los síntomas; sin embargo, este hallazgo no debe interpretarse como evidencia de un efecto protector ni como una participación específica relacionada con la menopausia, ya que la variable reflejaba la frecuencia y la duración del uso general de Internet y las redes sociales. Estos hallazgos subrayan la importancia de integrar la alfabetización en salud digital en el asesoramiento sobre la menopausia. Las enfermeras especializadas en salud de la mujer y otros profesionales de la atención de salud deben preguntar de manera proactiva sobre la búsqueda de información en línea, orientar a las mujeres hacia recursos confiables y apoyar la toma de decisiones informadas durante el período climatérico.

## Data Availability

Los datos que respaldan los hallazgos de este estudio están disponibles previa solicitud razonable a la autora de correspondencia.

## MATERIAL SUPLEMENTARIO

El siguiente material suplementario está disponible en línea:
